# A Putative Role of *Candida albicans* in Promoting Cancer Development: A Current State of Evidence and Proposed Mechanisms

**DOI:** 10.3390/microorganisms11061476

**Published:** 2023-06-01

**Authors:** Jasminka Talapko, Tomislav Meštrović, Branko Dmitrović, Martina Juzbašić, Tatjana Matijević, Sanja Bekić, Suzana Erić, Josipa Flam, Dino Belić, Anamarija Petek Erić, Andrea Milostić Srb, Ivana Škrlec

**Affiliations:** 1 Faculty of Dental Medicine and Health, Josip Juraj Strossmayer University of Osijek, 31000 Osijek, Croatia; 2 University Centre Varaždin, University North, 42000 Varaždin, Croatia; 3 Institute for Health Metrics and Evaluation and the Department of Health Metrics Sciences, University of Washington, Seattle, WA 98195, USA; 4 Department of Pathology and Forensic Medicine, University Hospital Center Osijek, 31000 Osijek, Croatia; 5 Department of Dermatology and Venereology, University Hospital Center Osijek, 31000 Osijek, Croatia; 6 Faculty of Medicine, Josip Juraj Strossmayer University of Osijek, 31000 Osijek, Croatia; sbekic@mefos.hr (S.B.);; 7 Family Medicine Practice, 31000 Osijek, Croatia; 8 Department of Radiotherapy and Oncology, University Hospital Center Osijek, 31000 Osijek, Croatia; 9 Department of Psychiatry, University Hospital Center Osijek, 31000 Osijek, Croatia

**Keywords:** *Candida albicans*, cancer, carcinogenesis, gastrointestinal cancer, oral cavity, skin cancer

## Abstract

*Candida albicans* is a commensal fungal species that commonly colonizes the human body, but it is also a pervasive opportunistic pathogen in patients with malignant diseases. A growing body of evidence suggests that this fungus is not only coincidental in oncology patients, but may also play an active role in the development of cancer. More specifically, several studies have investigated the potential association between *C. albicans* and various types of cancer, including oral, esophageal, and colorectal cancer, with a possible role of this species in skin cancer as well. The proposed mechanisms include the production of carcinogenic metabolites, modulation of the immune response, changes in cell morphology, microbiome alterations, biofilm production, the activation of oncogenic signaling pathways, and the induction of chronic inflammation. These mechanisms may act together or independently to promote cancer development. Although more research is needed to fully grasp the potential role of *C. albicans* in carcinogenesis, the available evidence suggests that this species may be an active contributor and underscores the importance of considering the impact of the human microbiome on cancer pathogenesis. In this narrative review, we aimed to summarize the current state of evidence and offer some insights into proposed mechanisms.

## 1. Introduction

The genus *Candida* spp. belongs to the microbiota of the oropharyngeal cavity, gastrointestinal tract, genital system, and skin [1]. Most of the species of this genus are opportunistic pathogens, and infections caused by them are primarily the result of disturbances in the balance of the normal microbiological flora caused by immunosuppressants, broad-spectrum antibiotics, or the loss of the protective epithelial barrier [2]. The principal risk factors for candidiasis are neutropenia following chemotherapy or immunosuppressive treatment (oncological, hematological, and transplant patients) [3], the use of broad-spectrum antibiotic therapy, the presence of a central venous catheter or urinary catheter, parenteral nutrition, renal dialysis, and the use of endoprostheses [4]. The disseminated form of candidiasis can affect the heart, lungs, central nervous system, musculoskeletal system, peritoneum, liver, spleen, and gall bladder [5]. In changing environmental conditions, *C. albicans* has the ability to transform into chlamydospores that enable adaptation to different places and situations [6]. The cell wall of *C. albicans* consists of carbohydrates, lipids, and proteins. It plays a significant role in the biology and pathology of this fungus because it possesses various mechanisms of pathological action [7] (Table 1).

*C. albicans* is a dimorphic fungus due to its ability to switch between yeast and hyphal forms and grows well in the presence of oxygen; conversely, the species thrives less in conditions with little oxygen and low redox potential [13]. Forming biofilms on inorganic and organic materials is crucial for its survival in the environment [14]. The hydrophobicity of the *C. albicans* cell surface is higher in anaerobic conditions, which has an impact on biofilm formation [15]. It is important to note that *C. albicans* within the biofilm can be more than 100 times more resistant to the action of antimycotics compared to planktonic cells [16].

## 2. Method of Data Collection

The PubMed database was searched for the keywords *‘Candida albicans’*, ‘cancer’, ‘carcinogenesis’, ‘gastrointestinal cancer’, ‘oral cancer’, and ‘skin cancer’. Additional investigations were carried out in combination with virulence factors and the mechanisms of action of *C. albicans* in individual organ systems. The search was limited to scientific articles published in the last twenty years (from 1998 to 2023) and prioritized articles published during the previous five years. The decisive factor for the inclusion of the article was the relevance of the content, evaluated based on the text of the content of the article. Therefore, the manuscript includes different types of studies performed on animal models, case reports, case-control studies, cohort studies, comparative studies, meta-analyses, and systematic reviews. A total of 118 related articles were examined in this review.

## 3. Exploring the Links between *Candida albicans* and Cancer

Numerous studies have described *C. albicans* as an opportunistic pathogen that exploits the immunosuppressed state of cancer patients, primarily due to chemotherapy [17]. However, recent research shows that *C. albicans* may actually prompt cancer development through multiple mechanisms, such as inducing inflammation, inducing Th17 (helper T cells) responses, producing carcinogenic byproducts, and using molecular mimicry [18] (Table 2). As a result, these mechanisms collectively increase the host’s susceptibility to cancer, such as oral, stomach, colorectal, and other cancers [19].

### Potential Carcinogenic Mechanisms of C. albicans

**Figure 1:**
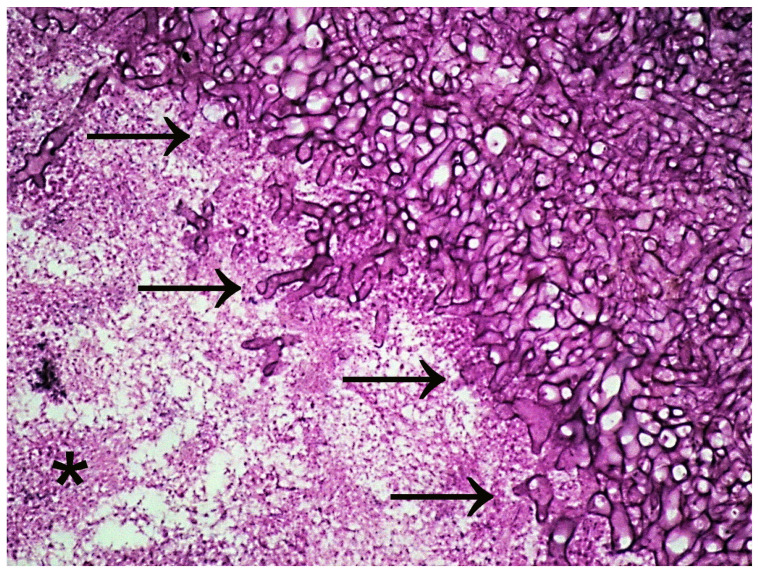
Histopathology of nasal polyposis showing classical PAS-positive budding fungal hyphae and spores (upper right-hand side arrows) admixed with eosinophils and neutrophils (asterisk). PAS (Periodic acid-Schiff) stains polysaccharide-laden fungal cell walls. Magnification 600×.

The first step of *C. albicans* colonization and its invasion is adhesion to mucosal epithelial cells (which are considered the first line of defense against microorganisms), weakening, in turn, the host’s defense mechanisms [34]. There is a significant role for a myriad of surface proteins—primarily adhesins, which enable the attachment of *C. albicans* cells to the mucosal wall [35]. The most important group of adhesins are Als agglutinin-like glycoproteins, most notably Als3 protein, which has been proven to play a role in adhesion to the oral and vaginal mucosa as well as in biofilm formation and induction of endocytosis [36]. Adhesins also include some Hwp hyphal wall proteins, such as Hwp1, which enable covalent attachment to cells since they are the substrate of transglutaminase from the surface of human cells [37]. Thus, the adhesins Als3 and Hwp1 mainly occur during the hyphae formation process of *C. albicans* and are key in adhesion to host cells [8] (Figure 1). Various factors influence the adhesion process, such as the type of proteins involved in the structure of the cell wall and the physicochemical properties of the cell surface [38]. After the integrity of the epithelial cells has been damaged, induced endocytosis occurs, followed by active infiltration, which is considered a pivotal step in pathogenesis [7,35]. The fungus’s weakening of the host’s defense mechanisms during colonization and invasion and the subsequent structural changes are linked with an increased risk of cancer development [19,20].

Moreover, the invasive enzymes possessed by *C. albicans* are of exceptional importance in the pathogenesis process, enabling it to destroy the integrity of the mucosal tissue structure and thereby contribute to the virulence of the strain and further impact on the cells [7]. *C. albicans* adhesins recognize and bind to ligands, such as proteins, fibrinogen, and fibronectin [39]. The outer membrane of human cells is a fluid phospholipid bilayer with incorporated proteins [40]. By secreting proteolytic enzymes that hydrolyze peptide bonds and phospholipases (i.e., enzymes that hydrolyze phospholipids), *C. albicans* penetrates through such a damaged membrane and can, subsequently, enter the cell [41]. This group of enzymes can also lyse microflora and, thus, reduce the host’s defenses [42].

Extracellular lipases of *C. albicans* facilitate adhesion to tissue and break down lipids, which are essential for collecting nutrients [42]. The most important are three groups of extracellular hydrolytic enzymes in the *Candida* species: aspartyl-proteinases, phospholipase B, and lipase [43]. Likewise, essential virulence factors include secreted aspartyl proteases (Sap) [44]. It is important to note that *Candida* expresses ten different Sap proteases, of which eight (Sap1–8) are free while two (Sap9 and Sap10) are bound to the cell surface [45]. Furthermore, there are four different classes of phospholipases (Pl), of which five are from class B (Plb1 to 5) extracellular and vital for virulence, alongside ten lipases (Lip1 to 10) [2,46]. Phospholipases hydrolyze host cell lipids, modeling their lipid substrate, making them crucial for virulence. During infection, an increased production of phospholipase B occurs, and its activity depends on changes in pH [47]. Depending on the Sap isoform, different clinical effects can develop, so Sap4, Sap5, and Sap6 are essential for systemic candidiasis while Sap1 to 3 are necessary for superficial mucosal and skin infections [48]. Thus, Sap proteases also participate in tissue penetration since their activity can directly damage collagen and other protein components of the tissue, phospholipases destabilize the cell membrane of epithelial and endothelial cells, and lipases modulate cell signaling between cells in their environment [7,49].

A biofilm formation is a significant factor in virulence [50]. The process starts when *C. albicans* cells adhere to the surfaces of a foreign body, forming a layer of cells and increasing the synthesis and production of hyphal wall proteins [51]. Then, there is active cell growth, extracellular matrix production, and hyphae formation that stabilizes the biofilm [52]. The *Candida* biofilm protects the cells from the immune system and antifungal therapy [53]. *C. albicans* also produces a vital virulence factor, the secreted hyphal-associated peptide toxin candidalysin Ece1, which is crucial for instigating mucosal and systemic infections. It has long been known that candidalysin is a hemolytic factor of *C. albicans* [54], which aids in obtaining nutrients for survival and reproduction [54]. In addition, candidalysin is vital in starting innate antifungal immunity during infection, largely dependent on neutrophil and interleukin 17 (IL-17) responses [55]. It is important to note that candidalysin can indirectly activate the epidermal growth factor receptor (EGFR), a complex mechanism involving EGFR and matrix metalloproteinase (MMP) ligands, which are involved in many types of cancer [56].

Phenotypic transformation helps *C. albicans* to adapt to its environment (in this case, the host tissue) [57]. When *C. albicans* invades epithelial mucosa cells, it can destroy the defensive barrier of the host epithelium, cause apoptosis or necrosis, and finally lead to structural changes in the epithelial cell [58]. This damages the epithelial cells and alters the normal structure, leading to abnormal proliferation associated with the development of cancer [59]. In addition, the ability of *Candida* to form biofilms can lead to a long-term exposure of host tissues to fungal carcinogens, such as acetaldehyde and an increased production of hydrolases, which can impair the innate immune system and cause chronic inflammation [19]. Risk factors for cancer development include chronic inflammation caused by microbial infections [60].

A chronic inflammatory condition can be caused by infections and permanent colonization of the mucosal epithelium [61]. *C. albicans* recognizes Toll-like receptors (TLRs) and C-type lectin-like receptors (CLRs), which activate the corresponding MAPK (Mitogen-Activated Protein Kinase) and NF-κB (Nuclear Factor kappa B) [62] (Table 3). Namely, the inflammatory signaling pathway and interferon promote the expression of manifold-related inflammatory genes. They are essential in developing malignant and benign conditions [19]. More specifically, cyclooxygenase enzymes (COX), MMPs, and prostaglandins can inhibit tumor suppressor genes through DNA methylation and post-translational modification, which potentially results in cancer development [63].

## 4. *Candida* Skin Colonization and Cancer Development

Skin cancer is the most frequently diagnosed malignancy in humans, and it is commonly divided into two subgroups: melanoma and nonmelanoma skin cancer (NMSC). NMSC or keratinocyte carcinomas are predominant ones, with basal cell carcinoma diagnosed in 75–80% and squamous cell carcinoma (SCC) in up to 25% of cases [66]. Although certain environmental risk factors (such as ultraviolet, ionizing radiation, and immunosuppression) are well established, others have yet to be explored [67]. For example, studies found an association between novel immunomodulatory therapy, including BRAF inhibitors and “biologic” therapy, with increased risk for skin SCC and keratoacanthomas [68,69,70]. Recently, there has been a special focus on researching the role of human microbiota in various human diseases, so naturally, the question of a potential link between skin microbiota and skin cancers was raised. Most studies have researched the association between bacteria and SCC, but studies identifying the role of fungal microbiota and fungal infections in skin cancer are deficient [71]. A chronic fungal skin infection has been recognized as a potential risk factor for skin SCC development. For example, one study reported about seven cases of skin SCC are derived from chronic chromoblastomycosis [72]. Regarding commensal fungi, limited studies implicate the protective role of *Malassezia* in SCC development [71].

*Candida* spp. are commensal fungi that colonize the skin of most healthy individuals. *Malassezia*, *Penicillium,* and *Aspergillus* spp. are more common, but the skin microbiota depends on the specific body site [73]. In addition, during certain conditions, such as antibiotic treatment and primary immunodeficiencies, fungal diversity increases, particularly of the *Candida* species [74,75,76]. The frequency of *Candida* species colonizing the skin differ from those in the human gut. Although *C. tropicalis, C. parapsilosis,* and *C. orthopsilosis* are species mostly detected on the skin, *C. albicans* most commonly causes symptomatic skin infections [1,73,75,77]. The most common site of *Candida* skin infections are intertriginous zones (e.g., submammary, inguinal folds, intergluteal crease, and pannus folds in the overweight patient). *C. albicans* can also cause chronic or acute paronychia, onychomycosis, genital infections, diaper dermatitis, and angular cheilitis. *C. albicans* usually causes superficial skin infections, and the involvement of deeper tissues in immunocompetent individuals is rare [1]. However, with medical progress, there is an escalating population of high-risk individuals, such as immunosuppressed patients, oncological patients, and transplant recipients. As a result, *Candida* skin infections are becoming more frequent and have a more protracted course [78].

Although many studies associate *Candida* with oral, esophageal, gastric, and colorectal cancer, as described previously, studies researching the association between skin cancer and a *Candida* infection or skin colonization are lacking. There are cases of verrucous candidiasis that progressed to moderate or severe dysplasia, but only one published case of verrucous candidiasis progressed to SCC [33]. Furthermore, only two population-based studies included skin cancer in evaluating cancer risk in patients with a *Candida* infection. The first nationwide study in Denmark did not find an increased risk for melanoma in such patients; however, patients with nonmelanoma skin cancer were not included in the study [79]. A second similar study was conducted in Taiwan, and they followed the occurrence of melanoma and nonmelanoma skin cancer separately. Results revealed a significantly higher risk of nonmelanoma skin cancer among patients with a *Candida* infection than those without [32]. These studies showed a possible association between a *Candida* infection and skin cancer, but the causal relationship is yet to be proven. This association could be possible because a *Candida* infection and skin cancer share common risk factors, such as immunosuppression and organ transplantation [80,81,82]. Therefore, more studies with improved methodology are needed to identify the role of *Candida* in the development of NMSC.

## 5. *Candida albicans* in the Context of Gastrointestinal Carcinomas

### 5.1. Candida albicans and Gastric Cancer

Gastric cancer is a prevalent disease worldwide, ranking as the fifth most pervasive cancer and presenting with a rather high mortality-to-incidence ratio as the third most frequent cause of death worldwide [83,84]. With the advent of high-throughput sequencing technology, appraising the link between the gastric microbiome (beyond the well-established *Helicobacter pylori*) and gastric cancer became a research priority. Recently, Zhong et al. analyzed metagenomic sequences from cancerous and adjacent non-cancerous tissues in 45 patients with gastric cancer to investigate the ecological changes within the gastric microbiome [85]. The results revealed that fungal communities in individuals with gastric cancer were unbalanced, with shifts in fungal composition and a large increase in the abundance of *C. albicans*. This led to the conclusion that this specific fungal species could indeed be a biomarker for gastric cancer [30].

Interestingly, an increase in *C. albicans* abundance in gastric patients from this study coincided with a corresponding increase in the abundance of other fungal species, such as *Arcopilus aureus*, *Fusicolla acetilerea,* and *Fusicolla aquaeductuum*. In contrast, *Aspergillus montevidensis*, *Candida glabrata*, *Penicillium arenicola,* and *Saitozyma podzolica* were found to be significantly decreased [85]. Furthermore, *C. albicans* was also implicated in depleting the abundance and diversity of other gastric fungi, which could potentially promote the rise of cancer in the stomach [85]. A recent report described a gastric cancer patient who had recently undergone cholecystectomy and presented with a *C. albicans* empyema that necessitated drainage and fluconazole treatment—underscoring the need to consider *C. albicans* in the pleural effusions of patients with malignancies [86].

In any case, the alterations in the gastric microbiome could be linked to the pathogenesis of gastric cancer and may even be seen as a biomarker of malignancy with potential clinical utility; however, the exact mechanism by which *C. albicans* contributes to gastric cancer progression on a molecular level remains unclear and underscores the need for further investigation [87].

### 5.2. Candida albicans and Colorectal Cancer

The latest estimates of the Global Burden of Disease (GBD) revealed 2.17 million cases and 1.09 million deaths in one year due to colorectal cancer [88]. Previous research has already shown that the intestinal microbiome could have an indispensable role in the development of this prevalent malignancy [89]; in addition, studies have revealed that an altered fungal network could be a contributing factor to the pathogenesis of colorectal carcinoma [19]. Although these findings do not have concrete clinical implications at the moment, this should be recognized as a potentially compounding factor in disease development.

A study that analyzed the biodiversity of intestinal microbiota showed that noteworthy alterations of mycobiota (with an overall lower diversity) might play a deciding role in the development of both polyps and colorectal carcinoma [90]. Another ecological analysis of gut microbiota reached analogous conclusions by demonstrating distinct mycobiomes in the early and late phases of the cancerous state as well as an increased Basidiomycota: Ascomycota ratio [91]. However, these research endeavors did not elucidate the circumstantial role of *C. albicans* and symbiotic fungi in the development of colorectal carcinoma [19].

Recent experiments demonstrated that the deletion of the Dectin-3 gene led to a substantial increase in colorectal cancer development, with a fungal burden in the feces of knockout mice being much higher when compared to wild-type mice [31]. Dectin-3 (also known as Cled4d or CLECSF8) is a type of pattern recognition receptor (PRR) primarily expressed on the immune cell surface and involved in the recognition and binding of fungal pathogens, such as *C. albicans* [92]. In the study by Zhu and co-authors (2021), the abundance/proportion of *C. albicans* has been significantly increased in knockout mice characterized by a deleted Dectin-3 gene. Furthermore, there was a positive correlation between the frequency of IL-22 in the tumor tissues of patients with colon cancer and the *C. albicans* burden [31], which suggests the significance of this regulatory pathway in human diseases.

Further experiments from this group involved transplanting feces from knockout, cancer-bearing mice into other mice, which confirmed that the feces and *C. albicans* could promote the process of colorectal carcinogenesis [31]. Interestingly, it was also described that antifungal treatment could relieve the tumor load of mice with a deleted Dection-3 gene and that the absence of this gene can hinder the propensity of macrophages to clear *C. albicans* from a certain site in the intestines [31]. Hence, the findings from these studies unveil the molecular process through which *C. albicans* regulates the intestinal immune system and contributes to the progression of colorectal cancer, but further studies are warranted to confirm this association.

In the interim, a conventional culture-based study from rectal swabs by Starý and co-authors (2020) can provide some ideas, as they have demonstrated a noteworthy and significant association of *C. albicans* with colorectal carcinoma cases; conversely, no such association has been established for cultivable bacteria. Potential confounding factors were accounted for, and the observation these authors made serves as a compelling motivation to validate the potential value of the readily cultivable *C. albicans* as a potential screening tool for individuals who are at risk of developing colorectal cancer or are in the early stages of the asymptomatic disease [93]. This should also promote the broader adoption of traditional culture methods and the advancement of more sophisticated culturomics techniques as supplementary strategies to (increasingly advocated, but still expensive) metagenomics [28]. In addition, the affordability and established standardization of traditional culture make it especially appropriate for conducting long-term studies that enable the gathering of extensive patient groups.

### 5.3. Candida albicans and Esophageal Cancer

Esophageal squamous cell carcinoma is more frequently observed in patients with long-standing esophageal *Candida* infections, particularly *C. albicans* [27,94]. The exact mechanism fundamental to esophageal carcinogenesis in individuals with chronic candidiasis is not fully understood, but it is believed to involve multiple factors. One important risk factor for the increased incidence of cancer is the production of nitrosamines related to *Candida,* and there is also a role of signal transducer and activator of transcription 1 (STAT1) defects [94]. There is also an issue of varying degrees of T-lymphocyte dysfunction in patients with *C. albicans* that can predispose patients to cancer.

A case report from Brazil highlights the development of epidermoid esophageal cancer after harboring treatment-refractory esophageal candidiasis for decades [29]. One report from the Netherlands presents two cases of individuals with chronic esophageal candidiasis who subsequently developed esophageal carcinoma [94]. One of the patients was diagnosed with autosomal dominant chronic mucocutaneous candidiasis, which was attributed to mutations in the DNA sequence that encodes the STAT1. The second patient had refractory esophageal candidiasis for more than a decade, with no other indications of chronic mucocutaneous candidiasis [94]. Another case series from the United Kingdom described six patients with carried a gain-of-function STAT1 mutation (GOF-STAT1) which predisposed them to inherited chronic mucocutaneous candidiasis—two of which developed and died of squamocellular esophageal carcinoma [95]. However, although the speculated mechanisms of cancer development are credible, this link is still tenuous and anecdotal.

In any case, the high risk of malignancy in patients with chronic candidiasis underscores the need for close monitoring to detect esophageal carcinoma at an early stage of development. It is pivotal to identify and manage risk factors to prevent the occurrence and progression of malignancies in patients with chronic candidiasis. Still, it is important to note that the presence of *C. albicans* in the esophagus does not necessarily indicate a direct causative relationship with esophageal cancer, which is important from the clinical vantage point. Other risk factors, such as tobacco and alcohol use, gastroesophageal reflux disease, obesity, and certain dietary factors, are known to play a significant role in the development of this type of cancer [96].

## 6. The Role of *Candida* in Oral Malignancy

The most common cancer observed in the oral cavity is planocellular carcinoma, also known as oral squamous cell carcinoma, which comprises over 90% of oral cancers [97,98]. It can develop in various sites of the mouth, including the tongue, buccal mucosa, lips, gingiva, or palate. In more than half of cases, oral planocellular carcinoma develops from premalignant lesions characterized by the appearance of white deposits or red lesions on the mucosa (leukoplakia/erythroplakia). Unfortunately, a prognosis and five-year survival rates are not particularly good, and 50% of cases end lethally [99,100].

**Figure 2:**
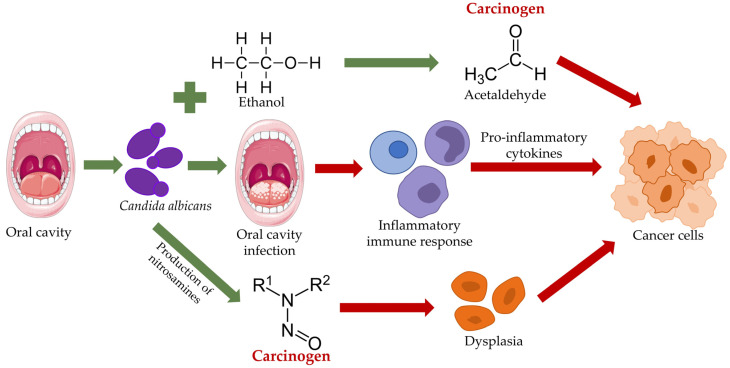
An overview of the various pathways by which *Candida albicans* may contribute to the development of oral cancer.

The relationship between *Candida* spp. and oral carcinogenesis has been a topic of discussion for a long time. Various possible ways in which this fungal genus may interact with the development of oral cancer have been suggested [26,47]. Several mechanisms that have been proposed are the induction of genetic instability in oral cells, promotion of epithelial cell transformation, carcinogenic byproducts, immune modulation, and chronic inflammation [19,20]. A recent systematic review concluded that the existence of a hallmark species of the genus, *C. albicans,* in the oral cavity could be linked to the development of oral cancer due to the changes it causes in the phenotypic structure of the cell, but also as a result of genotypic modifications [101]. Additionally, it was emphasized that *C. albicans* produces carcinogenic substances that can promote oral cancer progression [101] (Figure 2).

Research suggests that *C. albicans* can directly promote the development of oral cancer by producing certain carcinogenic enzymes and other substances that can damage DNA and promote the growth and survival of cancer cells. Some specific compounds in that realm are nitrosamines and acetaldehyde [102,103]. The ability of oral *Candida* to metabolize alcohol into acetaldehyde has proven to be very important. Acetaldehyde is known to cause DNA damage and inhibit DNA repair mechanisms, leading to genetic mutations and chromosomal abnormalities associated with cancer development [25]. This substance also binds indirectly to the important antioxidant glutathione, which increases the presence of reactive oxygen species, promotes chronic inflammation, and causes mitochondrial damage [26,104]. In addition, infection with *C. albicans* can suppress the immune system, making the host more susceptible to developing cancer. Finally, this fungus can also interfere with apoptosis or programmed cell death, which generally helps remove damaged or abnormal cells from the body, allowing them to continue proliferating and potentially leading to cancer [105].

This is in agreement with some recent findings. A study from Hungary demonstrated how *C. albicans* enhances the proliferation, migratory processes, as well as invasion of oral squamous cell carcinoma cells in laboratory conditions and also promotes tumor growth and metastases in test animals [20]. These authors observed that *C. albicans* infection increases gene expression pertinent to cell cycle regulation, frank inflammation, and epithelial-mesenchymal transition in cancerous cells. Such findings suggest that *C. albicans* may contribute to the progression of oral squamous cell carcinoma (both in early and late stages) through the modulation of tumor cell behavior and the host immune response by upregulating oncogenes and potentiating a premalignant phenotype, which has substantial implications for clinical practice [20].

Wang et al. aimed to investigate the underlying mechanism of how *C. albicans* promotes oral cancer from the vantage point of the tumor immune microenvironment [55]. They have discovered that a *C. albicans* infection enhances the expression of interleukin-17A (IL-17A) and its receptor (IL-17RA) in oral cancer cells and macrophages. Consequently, the increased IL-17A/IL-17RA signaling activates macrophages and promotes the release of inflammatory cytokines, which in turn enhances the proliferation, migration, and invasion of oral cancer cells [21]. In addition, the study shows that *C. albicans*-induced IL-17A/IL-17RA signaling promotes the growth of oral cancer xenografts in vivo [21].

There is further evidence for this association. By using single-cell expression profiling, Hsieh et al. found that immune cell infiltration can be observed in carcinogenesis prompted by a *C. albicans* infection and elucidated comprehensive mechanisms that underpin such processes [22]. Wang and colleagues established how *C. albicans* upregulates the expression of programmed death-ligand 1 (PD-L1) in oral cancer cells both in vitro and in vivo, leading to an inhibition of T cell activation and proliferation [23]. Moreover, Marin-Dett et al. (2023) found that a *C. albicans* biofilm may contribute to the development and progression of oral cancer by inducing lipid droplet formation and decreasing the efficacy of chemotherapy drugs [24]. Although there are no clear-cut conclusions for now, there is increasing evidence to suggest that the presence of this *Candida* species is indeed linked to a higher risk of oral squamous cell carcinoma development [106].

Another important virulence factor that may play a role in the influence of *C. albicans* on the development of oral cancer is candidalysin. Candidalysin is a protein toxin secreted by the fungus *C. albicans*, which is known to cause oral candidiasis, a common fungal infection in the mouth. The significance of candidalysin in the development of oral cavity carcinoma is not yet completely understood; still, some research suggests that it may play a role in the early stages of carcinogenesis [77]. Studies have shown that candidalysin can activate the immune system and promote inflammation, possibly contributing to cancer development. In addition, candidalysin has been found to interact with and disrupt oral epithelial cell membranes, which can lead to DNA damage and mutations [78]. In addition, candidalysin has been shown to promote angiogenesis and the formation of new blood vessels. Angiogenesis is a crucial step in the growth of cancer and metastasis of primary tumors in other tissue and organs, suggesting that candidalysin may contribute to the progression of oral cavity carcinoma. Overall, although more research is needed to fully understand the role of candidalysin in oral cavity carcinoma, it is clear that this toxin may play an important role in the development and progression of this disease [107,108].

It has to be emphasized that *C. albicans* normally colonizes the oral cavity of humans [109,110]. It is the most abundant yeast fungus on both the healthy and infected oral mucosa [1,109], which is why there is a need for further studies to establish the link in the chain of cancer development fully. Its conversion to a pathogenic state and the occurrence of opportunistic infections are the results of disturbances in homeostasis caused by various environmental and hereditary factors, which may be local or systemic [1].

It has long been known that *C. albicans* infection of the oral cavity is pervasive in oncology patients as a result of chemotherapy and radiotherapy and—as an opportunistic pathogen that takes advantage of the state of immunosuppression—has usually been associated with malignancies [18,111,112]. However, alongside the already mentioned studies, its role in initiating and developing oral cancer has recently been increasingly studied [18,26,47,101,111,113,114].

*C. albicans* infections in the oral cavity have a wide spectrum of manifestations; on this basis, there are several subdivisions of candidiasis. The color of the change in the mouth can be divided into red and white and, according to the origin, into primary and secondary. Primary candidiasis refers to infections that affect only the perioral area and oral cavity. However, when the infection occurs in the context of systemic diseases and the mucosa is already altered (and, thus, amenable to infection), it is referred to as secondary candidiasis [1,115]. Pseudomembranous candidiasis, acute erythematous candidiasis, chronic erythematous candidiasis, and chronic hyperplastic or nodular candidiasis are considered primary forms of the disease. Conversely, angular cheilitis, medial rhomboid glossitis, and chronic mucocutaneous candidiasis are secondary [1,109,115].

Chronic hyperplastic candidiasis, also known as *Candida* leukoplakia, has the highest rate of malignant transformations of the aforementioned *Candida*-induced oral lesions [101,112,116]. It is characterized by white deposits on the mucous membrane that cannot be easily removed with gentle scraping. This distinguishes it from other types of candidiasis and lesions that also appear in the form of white deposits. The reason for this is fungal hyphae’s deep infiltration of the oral cavity tissues. White deposits in the mouth can be homogeneous or heterogeneous, with the latter being particularly prone to malignant alteration. Changes on the oral mucosa are usually painless but may be sensitive to touch, spicy foods, or heat. They can occur anywhere in the mouth but are most common on the lateral parts, under the tongue, and on the buccal mucosa [1,115]. The exact cause of oral leukoplakia is unknown; nonetheless, it is thought to be related to a chronic irritation of the oral tissues, such as from smoking, rough teeth, or dental appliances. Treatment for oral leukoplakia usually involves eliminating the source of irritation, such as smoking cessation or replacing bad-fitting dental appliances and monitoring the patches for changes or growth over time. In some cases, a biopsy may be needed to determine whether the patches are cancerous or precancerous [117].

A *Candida* infection has been suggested to act as a co-carcinogen in the presence of other risk factors. For example, in individuals who smoke or consume alcohol excessively, a *Candida* infection may synergistically interact with these habits to increase the risk of developing oral precancerous lesions [101]. Although the importance of *C. albicans* in oral cancerogenesis is not fully understood, some evidence suggests that it may play a contributing role. Studies have shown that chronic inflammation, which can be caused by a *C. albicans* overgrowth, is a key factor in the formation of many cancers, including oral cancer. In addition, interactions between *Candida* and other groups of oral microorganisms may contribute to the development of specific oral diseases. For example, *Candida* has been found to interact with bacteria such as *Streptococcus mutans*, which is related to dental caries and *Porphyromonas gingivalis*, which is related to periodontal disease. These interactions can subsequently lead to the formation of mixed-species biofilms, which can increase the virulence of these microorganisms and contribute to the progression of severe oral diseases—including oral premalignancy and malignancy [47].

In a nutshell, individuals with chronic or recurrent *Candida* infections, particularly those with compromised immune systems or other risk factors for oral cavity cancer, may be prone to develop cancer of the oral cavity [1]. Maintaining a healthy oral microbiome through good oral hygiene and continuous dental visits can help prevent these conditions from developing. For these individuals, it is important to seek immediate medical attention for oral symptoms, such as white patches or sores, as well as treat the underlying risk factors to prevent the potential occurrence of oral cancer [118].

## 7. Conclusions

Notwithstanding the putative risk of malignancy suggested by many in vitro and in vivo studies, evidence linking *C. albicans* to cancer is still emerging. Thus more research is needed to establish a causal relationship. However, the potential mechanisms proposed in this review highlight the need for increased awareness of the impact of chronic fungal infections on cancer development—particularly in the oral cavity and the remainder of the gastrointestinal tract. Future studies should focus on identifying biomarkers of a *C. albicans* infection that reveal whether carcinogenesis started to occur, as that may prove to be a pivotal step in developing targeted interventions to prevent or treat *Candida*-associated malignant diseases. From a clinical research point of view, future studies should focus on prospective designs with long-term follow-up to evaluate the association between *C. albicans* infections and different carcinomas. These studies should also aim to account for confounding factors, such as other known risk factors for cancer, and consider the interplay between the host’s immune response, microbiota composition, and the presence of the fungus. For now, the putative link should be acknowledged, but it is pivotal to interpret all the available evidence with caution.

## Figures and Tables

**Table 1:** *C. albicans* mechanism of pathological action.

No. of Mechanism	Mechanism	Reference
1.	adhesion to different surfaces	[8]
2.	morphological changes	[9]
3.	adaptation to different environmental conditions	[1]
4.	production of hydrolytic enzymes	[10]
5.	biofilm formation	[11]
6.	avoidance of host defenses	[12]

**Table 2:** The association between *C. albicans* and cancer.

Cancer Type	Findings	Methods	Refs.
Oral cancer	*C. albicans* enhances the proliferation, migratory processes, as well as invasion of oral squamous cell carcinoma cells in laboratory conditions and also promotes tumor growth and metastases in test animals.	Modulation of tumor cell behavior and the host immune response by upregulating oncogenes and potentiating a premalignant phenotype.	[20]
	*C. albicans* infection enhances the expression of interleukin-17A(IL-17A) and its receptor (IL-17RA) in oral cancer cells and macrophages.	The increased IL-17A/IL-17RA signaling activates macrophages and promotes the release of inflammatory cytokines, which in turn enhances the proliferation, migration, and invasion of oral cancer cells.	[21]
	Immune cell infiltration was observed in carcinogenesis prompted by *C. albicans* infection.	Single-cell expression profiling	[22]
	Upregulation in programmed death-ligand 1 (PD-L1) expression in oral cancer cells.	Inhibition of T cell activation and proliferation by upregulation of programmed death-ligand 1 (PD-L1) expression in vivo and in vitro.	[23]
	*C. albicans* biofilm may contribute to the development and progression of oral cancer.	Induction of lipid droplet formation and decreasing the efficacy of chemotherapy drugs	[24]
	Genetic mutations and chromosomal abnormalities can be associated with the development of cancer.	DNA damage and inhibition of DNA repair mechanisms cause by acetaldehyde.	[25]
	Genetic mutations and chromosomal abnormalities can be associated with the development of cancer.	Reactive oxygen species promote chronic inflammation and cause mitochondrial damage.	[26]
Esophageal cancer	Development of epidermoid esophageal cancer.	Treatment-resistant esophageal candidiasis.	[27]
	Chronic mucocutaneous candidiasis leads to squamous cell carcinoma.	Mutation in STAT1 protein	[28,29]
Gastric cancer	An imbalance in fungal communities with changes in fungal composition and a large increase in the abundance of *C. albicans* leads to gastric cancer.	The increase in *C. albicans* is involved in the decrease in the abundance and diversity of other gastric fungi.	[30]
	Deletion of the Dectin-3 gene led to a substantial increase in colorectal cancer development, with fungal burden in the feces of knockout mice.	The deletion of the Dectin-3 gene led to a significantly increased abundance/proportion of *C. albicans* in knockout mice.	[31]
	Differences in the composition of the feces and abundance of *C. albicans* could promote the process of colorectal carcinogenesis.	Transplantation of feces from knockout, cancer-bearing mice into other mice confirmed that the feces and *C. albicans* could promote the process of colorectal carcinogenesis.	[31]
Skin cancer	Compared with the control group, patients with *Candida* infection had a significantly higher risk for overall skin cancer.	A case-control study enrolled 34,829 patients with Candida infection and an equal number of controls.	[32]
	Progression of verrucous candidiasis of lip to SCC after 12 months of follow-up.	A case report	[33]

**Table 3:** Purported mechanisms observed in *C. albicans*-induced cancer development.

No.	Mechanism	References
1.	activation of epithelial MAPK and ERK signaling pathways that are associated with growth and proliferation	[64]
2.	loss of E-cadherin and occluding observed in epithelial-mesenchymal transition (EMT)	[55]
3.	activation of angiogenesis and proangiogenic factors	[65]
4.	enhanced production of known carcinogenic molecules such as nitrosamines and acetaldehyde	[19,47]

## Data Availability

Not applicable.
